# Daily commuting to work is not associated with variables of health

**DOI:** 10.1186/s12995-016-0103-z

**Published:** 2016-03-22

**Authors:** Daniel Mauss, Marc N. Jarczok, Joachim E. Fischer

**Affiliations:** Occupational Health Services, Allianz SE, Königinstrasse 28, D-80802 Munich, Germany; Mannheim Institute of Public Health, Social and Preventive Medicine, Medical Faculty Mannheim, Heidelberg University, Ludolf-Krehl-Str. 7-11, D-68167 Mannheim, Germany; Institute of Medical Psychology in the Center for Psychosocial Medicine, University Hospital Heidelberg, Heidelberg, Germany

**Keywords:** Corporate health management, Employees, Heart rate variability, Psychosocial load, Stress, Work-life balance

## Abstract

**Background:**

Commuting to work is thought to have a negative impact on employee health. We tested the association of work commute and different variables of health in German industrial employees.

**Methods:**

Self-rated variables of an industrial cohort (*n* = 3805; 78.9 % male) including absenteeism, presenteeism and indices reflecting stress and well-being were assessed by a questionnaire. Fasting blood samples, heart-rate variability and anthropometric data were collected. Commuting was grouped into one of four categories: 0–19.9, 20–44.9, 45–59.9, ≥60 min travelling one way to work. Bivariate associations between commuting and all variables under study were calculated. Linear regression models tested this association further, controlling for potential confounders.

**Results:**

Commuting was positively correlated with waist circumference and inversely with triglycerides. These associations did not remain statistically significant in linear regression models controlling for age, gender, marital status, and shiftwork. No other association with variables of physical, psychological, or mental health and well-being could be found.

**Conclusions:**

The results indicate that commuting to work has no significant impact on well-being and health of German industrial employees.

## Background

Commuting to work daily is thought to be an important psychosocial risk factor associated with reduced physical and mental health of employees [1, 2]. Before the 19th century, most workers lived less than a one-hour’s walk from their workplace. Nowadays, many people commute a long way from their hometown, especially in industrialized societies. Modes of commute may include automobiles, motorcycles, public transport, bicycles, and walking. The prototypical commuter lives nearby one of the large cities surrounded by exurbs and travels daily to work. According to statistical explorations on 380.000 people in 2008, sixty-four percent of German employees commute to work by private car resulting in morning and evening rush hours and increased travel time over the last years [3]. Commuting is a widespread phenomenon and will increase over the next decades due to settlement patterns and availability of workplaces. The average daily commuting time in the former EU15 is 37.5 min and in the United States 48.8 min [4].

There is no standardized definition of work commute. One common used approach is daily travelling to work for more than 45 min one way [5]. Other authors propose the use of subcategories for near (<20 min.), middle (20–45 min.) and long commute (>45 min.) independent of travelled distance [1]. Commuting to work might probably affect well-being, characterized by satisfactory health and happiness, as well as physical and mental health of employees [6]. Nevertheless, there is only very limited evidence of large cohort studies available with most of them exploring self-rated variables of mental and physical health [7–9] as well as psychosomatic symptoms including gastrointestinal problems or heart palpitations [1]. Only very few authors have associated commuting with clinical assessed outcomes such as elevated pulse and blood pressure [10], or other indicators of the metabolic syndrome [11], as well as elevated salivary cortisol [12]. To investigate possible associations of commuting to work and physical health, variables should be clinically assessed which was almost never the case in the past.

The aim of this cross-sectional study was to explore the association of multiple health variables and work commute in industrial employees. We hypothesized that commuting to work would be negatively associated with self-rated and clinically assessed variables of well-being and health.

## Methods

### Study population

This report is based on cross-sectional data from the Mannheim Industrial Cohort Studies (MICS). Employees (*n* = 4881; 41.1 ± 11.5 years; 21.7 % female) of an industrial company in Southern Germany participated in a voluntary health risk assessment during working hours between September 2009 and May 2011. Of these, 3805 participants (41.1 ± 11.3 years, range 16–64 years, 21.1 % female) presented a full data set. The study was approved by the Medical Ethic Committee of the Medical Faculty Mannheim of Heidelberg University (approval number 2010-296E-MA). Written informed consent was given by each participant.

### Measurements

All data collection were conducted by an external agent (HealthVision Ltd, Berlingen, Switzerland). A comprehensive online health questionnaire included the question about minutes commuting to work one way. According to previous studies, commuting to work was grouped into four categories: 0–19.9, 20–44.9, 45–59.9, ≥60 min [1, 5]. Modes of commuting were unknown but likely by private car given travel patterns in Germany, especially in rural areas [3]. Sociodemographics (age, gender, marital status, single earner status, shiftwork), current smoking status, absenteeism and presenteeism (days per year) were assessed by self-report, as well as characteristics of well-being such as the 6-items mental and 6-items physical health subscales of the short form health survey (SF-12) (0–100, higher values = better health) [13], 5-items Cohen’s perceived stress scale (5–25, higher values = higher stress) [14], 6-items Maastricht Vital Exhaustion Questionnaire (6–30, higher values = higher exhaustion) [15], and 4-items Jenkins sleep scale (4–24, higher values = worse sleep quality) [16].

Clinical measurements assessed body mass index (kg/m^2^), waist circumference (centimeter), heart rate variability (root mean square of successive differences in milliseconds), diastolic and systolic blood pressure (mmHg). Fasting blood samples were collected between 7 and 9 a.m., immediately transported to a laboratory (Synlab, Augsburg Germany) and included following biomarkers: serum C-reactive protein, total cholesterol, high-density lipoprotein, low-density lipoprotein, triglycerides, glycosylated hemoglobin, and fasting plasma glucose.

Long term heart rate was recorded and beat-to-beat intervals were determined as the interval between two successive R-spikes and analyzed by researchers at the Center for Neuropsychological Research (University of Trier, Germany). Heart rate variability measures were calculated by usual means [17] as described elsewhere [18].

### Statistical analyses

We present descriptive, univariate analysis. Differences between the four commuting categories were determined by Student’s *t* test for continuous variables and by analysis of variance (ANOVA) for categorical variables. Bivariate associations were calculated using Pearson correlation. Linear regression models tested the association of commuting (independent variable) with multiple health variables (dependent variable), adjusting for age, gender, marital status, and shiftwork. Skewed variables were transformed according to the ladder of power to better approximate a normal distribution [19]. We used Stata 12.1 MP (College Station, TX: StataCorp LP) for all statistical analysis.

## Results

Mean travel time of the study sample was 29.0 (±18.9, range 1–150) minutes one way. Characteristics of the four subgroups of the study sample are presented in Table 1. Sample characteristics differed significantly across groups with respect to 12 of all 25 variables under study. The subgroup of short-commuters tended to be younger (39.5 ± 11.1 years), included more shift workers (11.7 %), and smokers (19.7 %) than participants of other subgroups.

**Table 1:** Characteristics of study sample (*n* = 3805)

	Short commute (*n* = 1133) <20 min	Middle commute (*n* = 1970) 20–44.9 min	Long commute (*n* = 363) 45–59.9 min	Very long commute (*n* = 339) ≥60 min	*p* value
Sociodemographics					
Age (years)	39.5 ± 11.1	42.2 ± 11.3	41.2 ± 11.5	40.5 ± 11.4	<0.001
Gender (% female)	21.9	19.0	30.3	21.2	<0.001
Marital status (% cohabitated)	69.5	75.2	76.6	74.0	0.002
Single earner (%)	78.3	78.4	73.4	76.8	n.s.
Shiftwork (%)	11.7	10.6	5.8	3.0	<0.001
Self-rated variables					
Current smoking (%)	19.7	17.8	17.1	18.0	n.s.
Absenteeism (days per year)					n.s.
0 (%)	18.4	17.4	17.6	19.8	
1–3 (%)	33.3	30.7	32.1	35.1	
4–10 (%)	24.0	24.0	24.3	23.1	
11–30 (%)	14.7	15.3	16.2	12.6	
>30 (%)	5.5	7.3	3.6	4.5	
Presenteeism (days per year)					n.s.
0 (%)	26.0	27.2	27.0	27.9	
1–3 (%)	25.9	23.6	25.6	21.9	
4–10 (%)	18.4	19.5	17.8	22.8	
11–30 (%)	13.8	14.9	12.8	12.0	
>30 (%)	9.1	8.3	10.0	9.3	
SF-12 mental health score (0–100)	47.5 ± 9.4	48.0 ± 9.3	46.8 ± 10.0	47.3 ± 9.8	n.s.
SF-12 physical health score (0–100)	53.0 ± 6.6	51.7 ± 7.2	52.0 ± 7.3	52.9 ± 6.5	<0.001
Perceived stress (5–25)	13.4 ± 3.8	13.6 ± 3.8	13.9 ± 4.0	13.6 ± 4.0	n.s.^a^
Exhaustion (0–36)	13.8 ± 4.8	13.9 ± 4.8	14.2 ± 5.1	14.0 ± 4.9	n.s.
Sleep quality (4–24)	5.1 ± 3.8	5.4 ± 4.1	5.3 ± 3.8	4.9 ± 3.7	n.s.^a^
Clinically assessed variables					
Systolic blood pressure (mmHg)	136 ± 13.9	136 ± 13.7	136 ± 14.7	135 ± 12.6	n.s.^b^
Diastolic blood pressure (mmHg)	78 ± 11.4	79 ± 11.2	79 ± 12.0	78 ± 11.2	0.035
Waist circumference (cm)	89.7 ± 11.5	91.7 ± 11.9	90.1 ± 12.7	91.3 ± 12.6	<0.001^c^
Body-mass-index (kg/m^2^)	23.2 ± 3.8	23.7 ± 3.8	23.2 ± 4.0	23.4 ± 3.8	0.002^b^
Fasting plasma glucose (mmol/l)	4.9 ± 0.7	4.9 ± 0.7	4.9 ± 0.6	4.8 ± 0.6	n.s.
Glycosylated hemoglobin (%)	5.6 ± 0.4	5.6 ± 0.4	5.6 ± 0.4	5.6 ± 0.4	n.s.
Total cholesterol (mmol/l)	5.5 ± 1.1	5.6 ± 1.1	5.6 ± 1.1	5.4 ± 1.1	0.018^a^
Triglycerides (mmol/l)	1.5 ± 1.0	1.5 ± 1.0	1.5 ± 1.0	1.3 ± 0.7	0.003^a^
Low-density lipoprotein (mmol/l)	3.2 ± 0.8	3.3 ± 0.8	3.2 ± 0.8	3.2 ± 0.9	0.005^a^
High-density lipoprotein (mmol/l)	1.5 ± 0.4	1.5 ± 0.4	1.5 ± 0.4	1.5 ± 0.4	0.020^c^
C-reactive protein, high-sensitive (nmol/l)	16.2 ± 33.3	16.2 ± 39.0	16.2 ± 37.1	15.2 ± 26.7	n.s.^c^
Heart rate variability (RMSSD in msec)	30.6 ± 13.7	29.6 ± 12.5	29.9 ± 12.7	30.4 ± 13.7	n.s.^c^

Continuous variables are expressed as mean ± standard deviation, categorical variables as number (percent). Differences between commuting categories were determined by using Student’s *t* test for continuous variables and ANOVA for categorical variables

*RMSSD* root mean square of successive differences

*n.s.* not significant

Transformation: ^a^ = sqrt, ^b^ = 1/sqrt, ^c^ = log

Commuting was positively correlated with waist circumference (*r* = 0.04, *p* < 0.05) and inversely with triglycerides (*r* = 0.04, *p* < 0.05). These correlations did not remain statistically significant in linear regression models controlling for multiple confounder (Table 2). Self-rated variables of well-being such as perceived stress, exhaustion, sleep quality, and mental health were not associated with commuting. All results did not change in subgroup analysis for both gender (data not shown).

**Table 2:** Linear regression analysis with commuting as independent variable, short commute (<20 min) as reference (*n* = 1133), adjusted for age, gender, marital status, and shiftwork

	Middle commute (*n* = 1970) 20–44.9 min	Long commute (*n* = 363) 45–59.9 min	Very long commute (*n* = 339) ≥60 min
Self-rated variables			
SF-12 mental health score	0.50	−0.55	−0.19
SF-12 physical health score	−0.88***	−0.71	−0.13
Perceived Stress	0.20	0.44	0.28
Exhaustion	0.07	0.30	0.24
Sleep quality	0.18	0.11	−0.13
Clinically assessed variables			
Systolic blood pressure	−0.92	0.32	−1.86*
Diastolic blood pressure	−0.03	0.36	−0.37
Waist circumference	0.75*	0.82	1.31*
Body-mass-index	0.19	0.13	0.19
Fasting plasma glucose	0.03	−0.40	−0.75
Glycosylated hemoglobin	−0.01	0.00	0.00
Total cholesterol	−0.74	0.07	−5.25*
Triglycerides	−2.43	3.39	−15.93**
Low-density lipoprotein	0.25	−0.86	−2.73
High-density lipoprotein	0.26	0.96	0.75
C-reactive protein, high-sensitive	0.05	−0.03	−0.03
Heart rate variability (RMSSD)	0.65	0.50	0.47

Untransformed coefficients are displayed, transformed variables showed similar *p* value patterns

*RMSSD* root mean square of successive differences

*** *p* < 0.001; ** *p* < 0.01; * *p* < 0.05

## Discussion

Although commuting to work showed a slightly bivariate positive correlation with waist circumference and a negative with triglycerides, we could not find a significant linear association with multiple variables of physical and psychological health in adjusted regression models.

We cannot confirm the results of others cross-sectional studies [8, 9]. Length of commute was associated with self-reported hypertension in 4715 rail road commuters in New York [8]. Most of the 6810 participants of an Australian study were driving a car to commute to work (69 %) which was associated with being overweight or obese [9]. Given reports of the German Federal Bureau of Statistics our sample should include at least 64 % of car commuters [3, 20] and therefore show similar results. An American study with 4297 adults showed that commuting distance was positively associated with body mass index, blood pressure in fully adjusted regression models but not associated with blood lipids and fasting plasma glucose and a composite factor of the metabolic syndrome [11].

We cannot confirm the commuting paradox described by Stutzer and Frey in 2004 [21] explaining that people with long journeys to and from work are systematically worse off and report significantly lower subjective well-being. Nevertheless, a recent Swedish study showed that satisfaction with work commute has a substantial influence on overall happiness [22]. Commuting was not associated with absenteeism, presenteeism, perceived stress, and other variables of well-being in our sample, while other authors have described associations with self-reported variables such as negative moods, greater illness and work absenteeism, and decreased life satisfaction [6]. To our opinion, the compensation for the burden of commuting including better leisure-time possibilities such as outdoor and physical activity as well as lower rents for housing in our sample might be sufficient to explore similar results between subgroups. Additionally, all of the included industrial sites are located in the periphery of smaller cities (<30,000 people) where people are used to travel longer distances than in major cities and public transport might not be available without spending significantly more time for the journey.

### Limitations

First, we did not assess modes of transport to commute to work (e.g. public transport, car, bicycle, walking) as well as commuting distance. Although the biggest stressor is travel time [20] and perceived loads are independent of use by public transport or private car [5], future studies should incorporate these information. While there is only limited evidence for negative effects of car commute, there are several studies reporting positive impact of active commuting like cycling or walking to work on employee well-being [23] and physical health [24, 25]. Second, the healthy worker effect may have resulted in an underestimation of the explored effects as well as the fact that more health-conscious people attend to screenings in general. Third, due to our cross-sectional study design we cannot draw any causal conclusion about the direction of observed associations or possible effects. Fourth, our results may have limited generalization due to our study sample of predominantly male workers within one country.

## Conclusion

Our results indicate that commuting to work is not associated with reduced health and well-being of German industrial employees. Different variables could possibly moderate a theoretical impact of commuting. This could have practical implications for employers and their offerings of corporate health promotion to reduce loads of employees. Longitudinal studies are needed to explore further any possible health effects of commuting.

## Abbreviations

ANOVA: analysis of variance
EU15: 15 European union member countries
HRV: heart rate variability
MICS: Mannheim industrial cohort study
RMSSD: root mean square of successive differences
SD: standard deviation
SF-12: 12-items short form health survey
